# Machine learning for enumeration of cell colony forming units

**DOI:** 10.1186/s42492-022-00122-3

**Published:** 2022-11-05

**Authors:** Louis Zhang

**Affiliations:** Department of Molecular Biosciences, College of Natural Sciences, University of Texas at Austin, Austin, TX 78713-8058 USA

**Keywords:** Colony forming unit, Enumeration, Image analysis, Blue/white screen, Colony count

## Abstract

As one of the most widely used assays in biological research, an enumeration of the bacterial cell colonies is an important but time-consuming and labor-intensive process. To speed up the colony counting, a machine learning method is presented for counting the colony forming units (CFUs), which is referred to as CFUCounter. This cell-counting program processes digital images and segments bacterial colonies. The algorithm combines unsupervised machine learning, iterative adaptive thresholding, and local-minima-based watershed segmentation to enable an accurate and robust cell counting. Compared to a manual counting method, CFUCounter supports color-based CFU classification, allows plates containing heterologous colonies to be counted individually, and demonstrates overall performance (slope 0.996, SD 0.013, 95%CI: 0.97–1.02, p value < 1e-11, r = 0.999) indistinguishable from the gold standard of point-and-click counting. This CFUCounter application is open-source and easy to use as a unique addition to the arsenal of colony-counting tools.

## Introduction

### Colony counting

Bacterial colony enumeration is useful in microbiology because of the prevalence of bacteria in the environment and their importance in human and animal sciences. Counting colony forming units (CFUs) provide quantitative information regarding the health of a specific bacterial species in a particular sample. Not only does this information hold broad relevance in fields ranging from antibiotic discovery to genomics, there is also a demonstrated need for rapid and accurate counts in the medical field, where a certain bacterial density threshold may aid in treatment decision-making [1]. In addition, the ability to rapidly and accurately count bacterial cells is important in the food industry, where the presence of bacterial cells in food products may be indicative of spoilage or contamination [2].

Although the manual counting of plates using a point-and-click approach is considered the gold standard for microbiological tasks, it is a tedious and labor-intensive process. Although commercially available products capable of automating the bacterial colony quantification are available, they are quite expensive, limiting their availability.

### Colony screening by color

Color-modifying reporter genes are frequently used in microbiology and genetics owing to their high sensitivity and ease of detection [3]. The majority of commonly employed reporter genes that induce visually discernible traits usually involve the introduction of fluorescence or a modification of the bacterial color. The best-known and most widely used of these is the lacZ gene of *E. coli*, which turns the bacteria into a blue color when grown in a medium containing an X-gal substrate. Other commonly used reporter genes include the green fluorescent protein from jellyfish, i.e., *Aequorea victoria*, red fluorescent protein from Discosoma sp., and luciferase from *Photinus pyralis*. [4]. Color changes induced by reporter proteins have been used to monitor a variety of cellular processes and interactions, including a promoter analysis, gene delivery, drug discovery screening, receptor/ligand characterization, and signaling pathway analysis [5–8].

Blue/white colony screens are frequently used during plasmid cloning, which is critical for understanding the structure, function, and evolution of genes [9] as well as for genetic, protein, and metabolic engineering [10]. However, achieving an effective transformation is a nontrivial task and is influenced by external factors such as the plasmid size, degree of DNA compactness, and methods of transformation [11]. As such, an optimization of the transformation protocols is frequently required before further study can proceed. Therefore, the ability to classify colonies by color may facilitate the optimization of the transformation conditions.

Reporter gene assays are also frequently used to provide quantitative data on the underlying biological processes. The CFU counts derived from blue/white screens represent key sources of information for mutation and phenotype analyses. For instance, the supF shuttle vector-based mutagenesis assay is a frequently used technique for assessing the frequency of damage-induced mutagenesis in human and bacterial cells, where distinct blue and white counts indicate the mutation frequency [12]. Similarly, CFU counts from blue/white assays have also been used to monitor horizontal gene transfer rates [13].

### Current approaches

Traditionally, CFUs have been manually counted. Over the past few years, this process has been streamlined using software such as ImageJ, which enables point-and-click colony counts. Although this method is more efficient and less error-prone, it still requires significant time and labor, which greatly limits the number of different samples that can be processed in a given study.

A variety of software solutions based on computer vision and machine learning technologies have recently surfaced. Various scripts have been developed using the ImageJ framework, which combines thresholding, binarization, and watershed segmentation. However, they require familiarity with basic image-processing concepts and the ImageJ user interface [14]. Current standalone approaches such as NIST’s Integrated Colony Enumerato employ an imaging station for image capture and a colony segmentation algorithm based on extended minima and thresholding [15]. ClonoCounter, another popular application, requires the user to input three independent parameters: the gray level, maximum colony size, and colony grayscale distribution. Although finding the appropriate parameters requires some experience, tips are provided to help speed up the process [16]. Open-source methods, such as OpenCFU, employ a score map to increment morphologically valid spaces over a range of global thresholds [17]. Other approaches implement top-hat filtering, Otsu thresholding, or a Bayesian classifier to probabilistically estimate overlapping colonies [18]. Image enhancement based techniques have utilized the high correlation between colonies and the local maxima within the Mellin spectra used in colony counting [19]. Colonies are counted in ScanCount, which utilizes the circular Hough image transform technique, requiring users to specify the minimum colony size and the shape of the plate [20]. Recent methods, such as AutoCellSeg, require users to select both small and large colonies as *a priori* ground-truth information. The algorithm then iteratively segments watersheds into colonies [21].

However, modern approaches are hindered by three issues: an inability to count plates with a high colony density, a failure to segment colonies along the plate boundary, and blindness to color in classifying the CFUs. These three issues are further exemplified in the following:

OpenCFU, a popular open-source cell-counting solution, uses a traditional computer vision approach by iteratively thresholding a plate image and incrementing morphologically valid blobs according to a strict set of predetermined parameters [17]. Although this allows for a high sample throughput, the strict reliance on predetermined morphology parameters often reduces the applicability of this approach because it is often restricted to an overly specific analysis of a given plate image with a tendency to miss colonies. Furthermore, OpenCFU requires extremely high-quality images (3024 × 4032) to accurately segment images with high CFU densities (> 2000 CFU/plate), which is a hidden constraint that reduces the utility of this approach.

In addition, many colony counting programs are unable to count colonies next to the plate border, opting to ignore the entire border region in their analysis [18, 21]. This limitation can diminish the precision of the generated analysis and systemically skew the generated counts to a value lower than expected. Although certain solutions, such as AutoCellSeg, offer users the ability to mitigate this loss by manually selecting missed colonies, doing so requires significant user input.

The current automated CFU software does not include the ability to classify CFU by color, making it only useful when users want to determine the total colony counts for plates containing homogenous colonies. Therefore, there are currently no viable automation methods for many biological assays that may require the counting of colonies when grouped by color. This limitation is likely due to the difficulty in extracting information from multiple differently colored colonies because it requires rough knowledge of the number of clusters and their respective attributes to avoid rejecting differently colored colonies as outliers. Although OpenCFU offers a similar color grouping functionality, based on the testing conducted in this study, it is unable to detect colonies of different colors residing in the same plate.

### Hypothesis

Given the aforementioned limitations of existing CFU segmentation and counting software packages, an efficient and accurate application assisting in the enumeration of bacterial colonies was developed in this study. Three key features were prioritized during the development of this solution: (1) the general ability to count bacterial colonies using equipment commonly used in laboratories, such as a backlight and a smartphone, (2) an algorithmic innovation to improve the fidelity of the segmentation; and (3) a dedicated function to segment colonies based on plate-specific colony properties.

## Methods

**Fig. 1:**
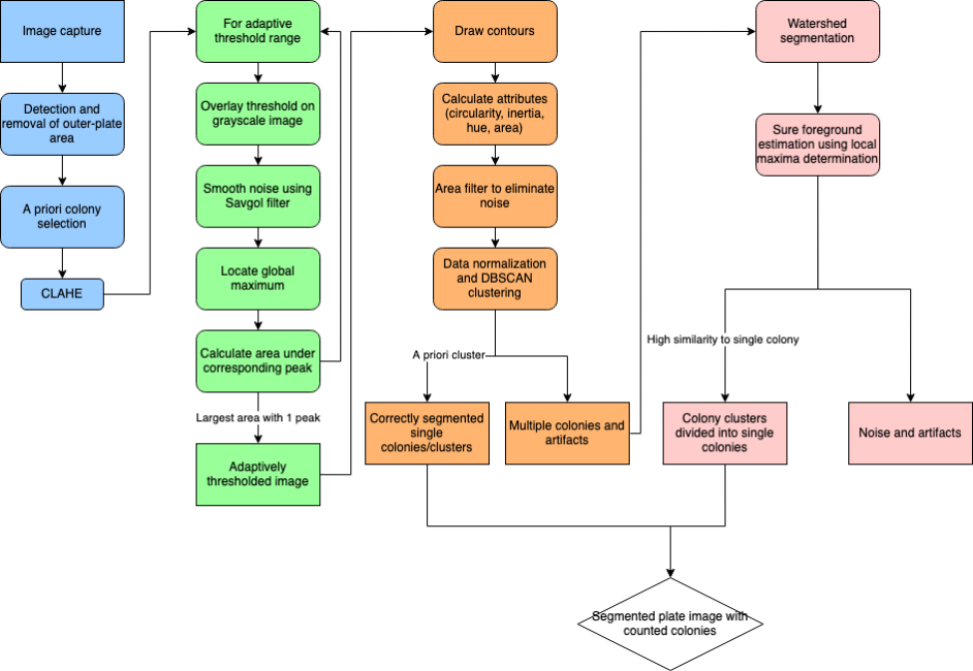
Flowchart of colony segmentation algorithm

A detailed flowchart depicting the algorithm is provided in Fig. 1.

### Bacterial culture preparation

*E. coli* cells were electroporated with plasmids and grown at 37 °C for 3 h. Half of the cells were plated on LB agar plates containing 50 µg/mL ampicillin, 24 mg/mL IPTG, and 20 mg/mL X-gal, whereas the other half were plated without X-gal. The plates were then incubated overnight at 37 °C and imaged.

### Hardware components

An iPhone 13 Pro was used to capture all petri dish images against a white light transilluminator.

### Image processing and segmentation

#### Image preprocessing methods

A preliminary mask of the plate edge was first generated, eliminating false-positive detections beyond the ROI. The resulting image was denoised using a median filter.

#### A priori colony identification

The user was prompted to click on a representative colony. From the clicked point, the colony was extracted through flood filling. All relevant attributes were calculated from the selected colonies and referenced as the ground truth.

### Image preproccessing

#### Iterative adaptive thresholding

A well-binarized image should clearly distinguish between the foreground and background. However, adequate binarization is a nontrivial task that must be sufficiently robust to function regardless of the grayscale value of the colony and variations in lighting. As a result, it is hypothesized that the grayscale histogram of a well-binarized image should meet three criteria: It should be unimodal and contain a large area under the peak, and the unimodal peak should include the previously determined grayscale value.

Because smartphone-captured images are highly susceptible to variable lighting, adaptive thresholding is applied. The image is initially divided into multiple subregions of user-defined size (s), with each subregion binarized independently using the following formula:

$$dst\left(x,y\right)=\left\{\begin{array}{c}0 if src\left(x,y\right)>T(x,y)\\ 255 if src\left(x,y\right)<T(x,y)\end{array}\right.$$

The threshold value *T*(*x*, *y*) is calculated as the difference between the Gaussian mean of each subregion and the user-defined value (C). Therefore, an effective binarization depends on the determination of these two parameters.

The grayscale histogram for each threshold iteration is calculated to determine the parameter combination that satisfies all three requirements. Owing to the noise inherent in grayscale histograms, each signal is smoothed using a Savitzky–Golay filter [22, 23]. The peak range is defined as the final value that continuously decreases from the maximum. To reduce the processing time, the area under the peak is calculated only if the mean *a priori* grayscale value is within the histogram peak range. When all three histogram conditions are satisfied, the combination of parameters that produces the largest area under the peak is chosen for further image processing.

The iteration ranges of s and C significantly affect the processing time. Therefore, the range is estimated using information derived from *a priori* colony selection. Because s must be larger than the size of the colony to avoid producing donut-shaped segments, the initial value is set to the argmax (width, height) of the enclosing bounding box of the *a priori* colony.

For images containing multiple colored colonies, distinct binary masks are generated in accord with the attributes of each user-selected colony and combined using a logical OR.

### Colony segmentation and clustering

The generated segments contain a mixture of single accurately segmented colonies, clusters of colonies, and artifacts. DBSCAN clustering and parameter filtering are used to identify individual colonies. The contour area, circularity, inertia, mean hue, and area ratio are evaluated. The eps parameter for DBSCAN is estimated using the kth nearest neighbor method, whereas the min_dist parameter is estimated as 1/5th of the total number of detected contours.

$$Circularity=\frac{4\pi *area}{{Perimeter}^{2}}$$

$$Inertia=\frac{Minor axis length}{Major axis length}$$

Among the various generated clusters, those containing the *a priori* colony/colonies are then further filtered through morphology cut-offs determined by an *a priori* selection to increase the strictness of the algorithm and minimize the likelihood of false-positive colonies. Because the detected single colonies are not further processed and serve as a guideline for further processing, post-DBSCAN filtering is required.

Segments that do not pass the strict single-colony test are considered to contain a mixture of irregular single colonies, overlapping colonies, and noise, which are then analyzed through local minima detection and a watershed transformation.

### Separation and verification of colony clusters

#### Watershed algorithm

The watershed algorithm is a classic approach in the computer vision field, which finds the largest connected subregions that are contained within a larger region. However, the watershed algorithm is susceptible to an oversegmentation owing to noise, and as a result, many computer vision packages, such as OpenCV, implement a marker-based watershed technique. Typically, these markers are generated through morphological erosion or distance transformations. However, because both techniques expect roughly uniform sizes of the subsegments, they have limitations that restrict their functionality for colony counting. To address this problem, a local minima detection algorithm was adopted that more closely resembles how a human will distinguish between adjacent colonies.

#### Local minima detector

Because circular bacterial colonies frequently have convex elevations, the centers of the bacterial colonies are typically darker than the surrounding regions. Capitalizing on this characteristic allows for a more robust sure-foreground capture through the detection of the local minima. The local minima are detected using the findmaxima2d package [23].

#### Secondary pruning of contours

**Fig. 2:**
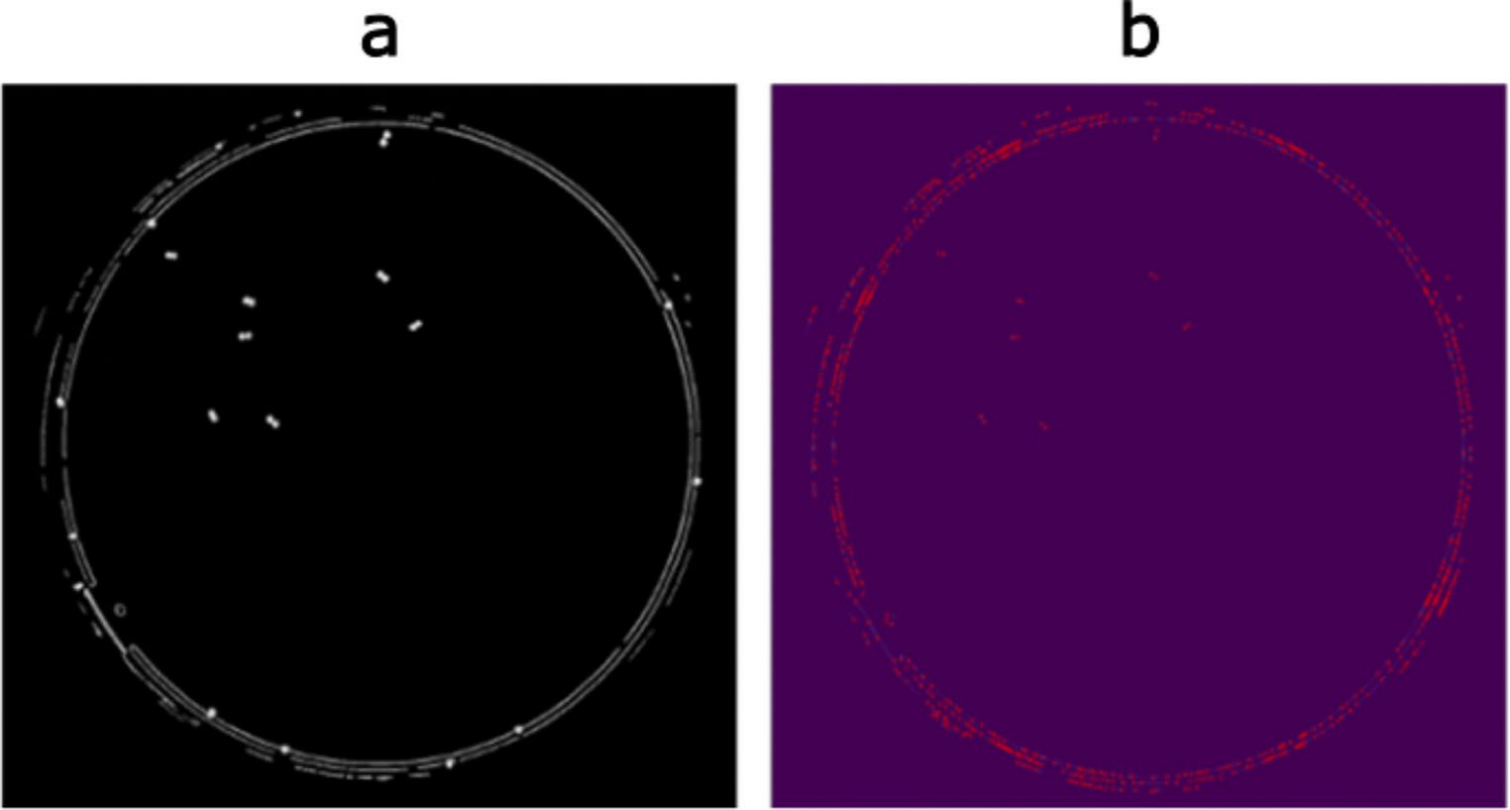
Overview of local minima detector. **a** A binarized inverted mask of a grayscale plate image containing only those regions that have been deemed as noise/colony clusters; **b** The local extrema detected. Note the high amount of noise from the plate borders that will need to be pruned through a comparison with the ground truth

As illustrated in Fig. 2, the sure foreground produced by the local minima detector contains both accurately detected colony centers and a significant number of artifacts. To differentiate between the two groups, the resulting contours generated from the watershed are filtered based on their mean HSV hue, circularity, inertia, and area, using values derived from the previously determined single colony cluster(s).

## Results

### Accuracy

To assess the accuracy of CFUCounter, its results were compared to those obtained manually using a point and click tool in ImageJ (the gold standard) and two leading CFU segmentation software solutions (OpenCFU and AutoCellSeg).

**Fig. 3:**
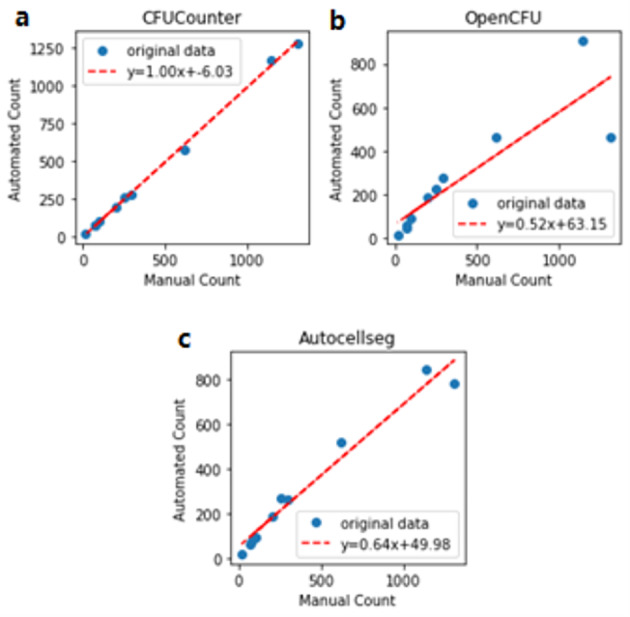
Linear regression of CFU counts using each method compared to the true number of CFU. **a** Accuracy of cell counter compared to the ground truth (slope 0.996; SD 0.013; 95%CI: 0.97–1.02; p value < 1e-11); **b** Accuracy of OpenCFU compared to the ground truth (slope 0.515; SD 0.101; 95%CI: 0.316–0.713; p value < 0.001) **c** Accuracy of AutoCellSeg compared to the ground truth (slope 0.638; SD 0.042; 95%CI: 0.555–0.721; p value < 1e-6)

All three methods were tested on 11 plate images containing either blue or white *E. coli* or Staphylococcus aureus, with a mean CFU count of 408.2 and a median of 228. The photographs were captured using a wide range of resolutions, from 1280 × 720 to 4032 × 3024. Four of the plate images contained blue *E. coli* cell colonies grown in media containing X-gal, three contained white colonies grown in standard media, and four contained *S. aureus *colonies obtained from the OpenCFUs plate image dataset. Among the three methods, CFUCounter produced the most accurate count relative to manual counting, with a slope of 0.996 (SD 0.013; 95%CI: 0.97–1.02; p value < 1e-11; r = 0.999) (Fig. 3a). AutoCellSeg performed the second best with a slope of 0.638 (SD 0.042; 95%CI: 0.555–0.721; p value < 1e-6; r = 0.983) (Fig. 3b). OpenCFU performed the worst with a slope of 0.515 (SD 0.101; 95%CI: 0.316–0.713; p value < 0.001; r = 0.874) (Fig. 3c). The slope of CFUCounter was also significantly different from that of OpenCFU (t value 4.70; p value 0.0001) and AutoCellSeg (t value 8.11; p value < 0.00001).

### Plate border analysis

**Fig. 4:**
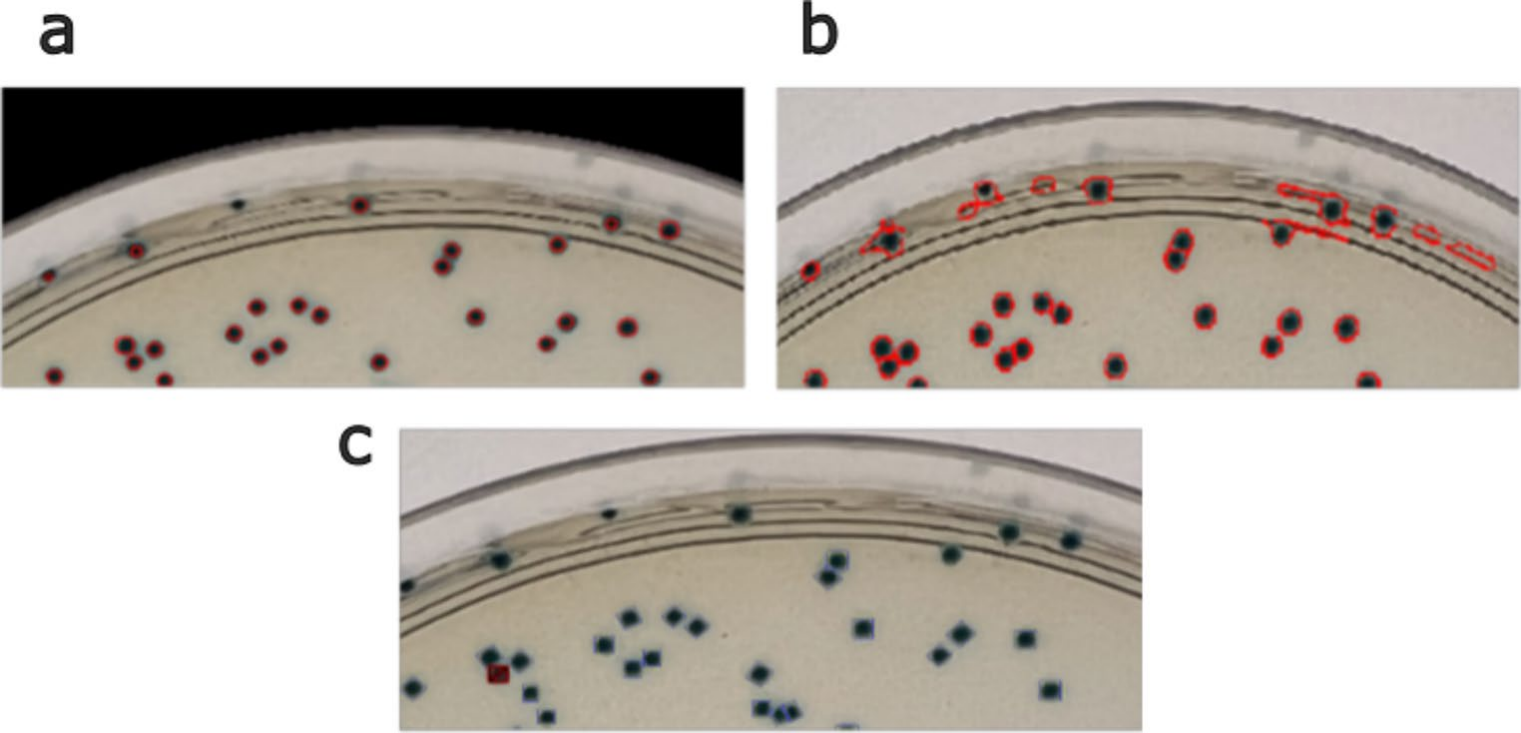
Comparison of plate border colony detection. **a** Segmentation results of plate border captured using CFUCounter; **b** Segmentation results of plate border captured using AutoCellSeg; **c** Segmentation results of plate border captured using OpenCFU.

Among the three methods tested, CFUCounter accurately segmented the cell colonies along the plate border. As illustrated in Fig. 4, CFUCounter correctly detected all colonies apart from the one along the edge, and OpenCFU was unable to detect any cells that touched the plate border. However, AutoCellSeg had the opposite issue of detecting a wide range of erroneous colonies (Fig. 4).

### Robustness

**Fig. 5:**
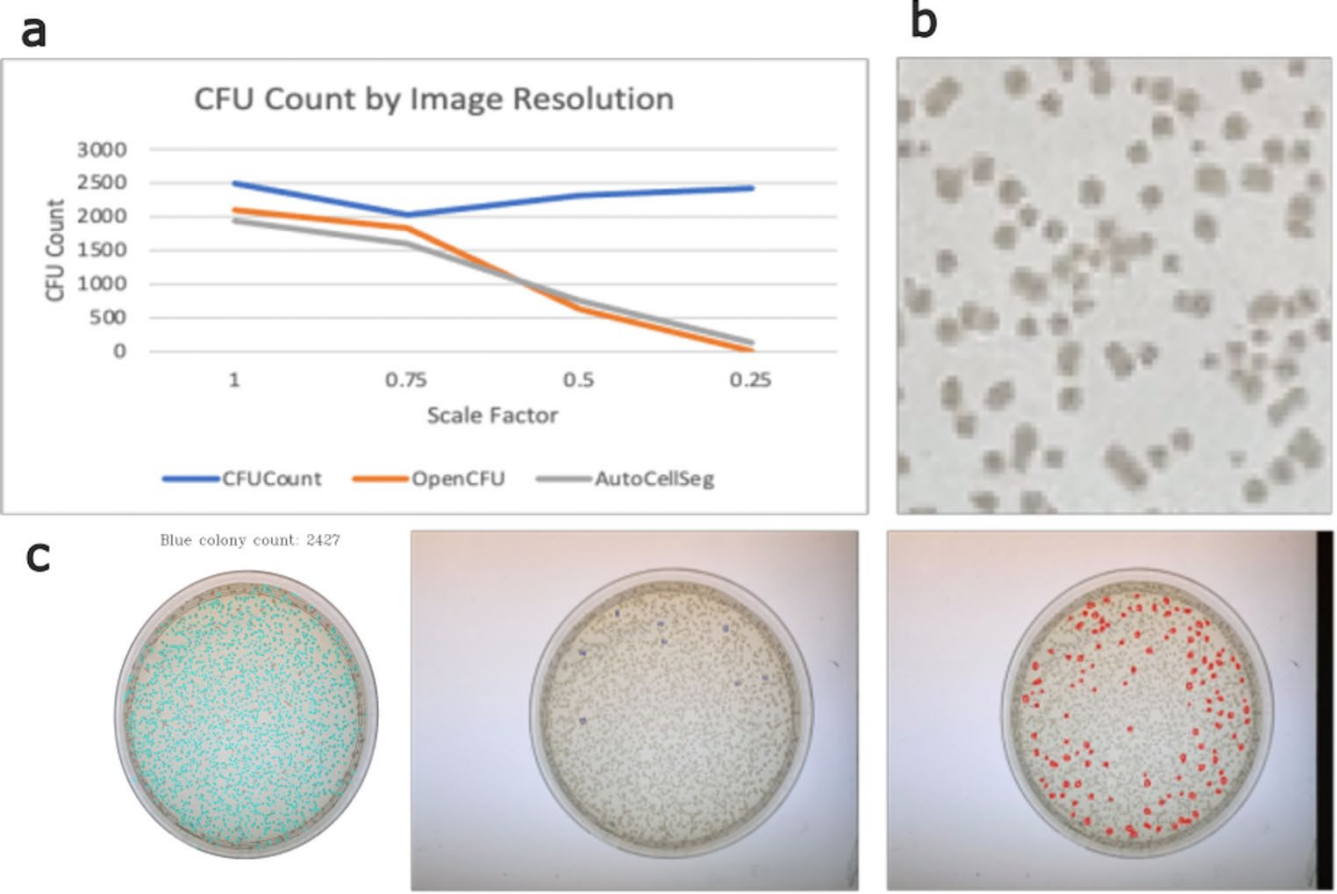
Impact of image resolution on colony segmentation. **a** Effect of image resolution on CFU count produced through each method. CFUCounter, OpenCFU, and AutoCellSeg were run on a plate image scaled by a factor of 1, 0.75, 0.5, and 0.25; **b** Zoomed-in view of the 0.25-scaled plate. Note the high amount of compression noise distorting the colonies; **c** Segmentation results of the 0.25x-scaled image. From left to right: CFUCounter, OpenCFU, and AutoCellSeg.

To assess the robustness of the three methods to the image resolution, a qualitative study was conducted to examine the colony counts produced through a 4032 × 3024 sized image when scaled by a factor of 0.75, 0.50, and 0.25. Although all three methods perform similarly for full-sized images, the counts produced by OpenCFU and AutoCellSeg decrease precipitously as the image resolution declines (Fig. 5a).

### Multiple color detection

**Fig. 6:**
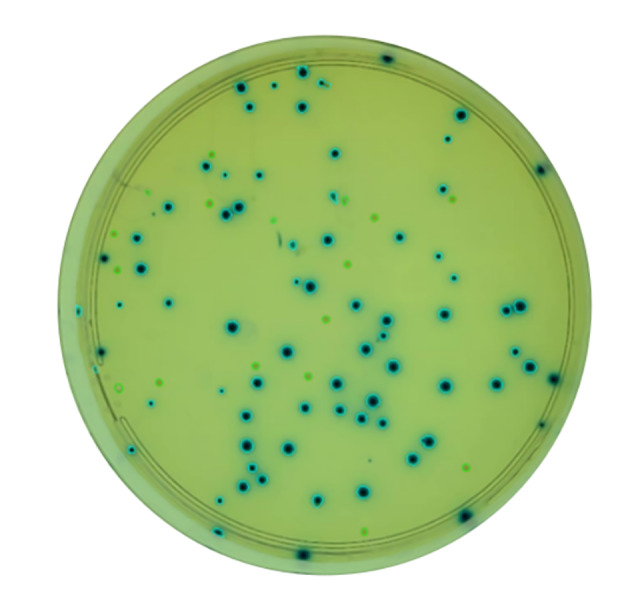
Segmented plate containing blue and white *E. coli* colonies. White colonies are circled in green and blue colonies are circled in blue

Because AutoCellSeg does not include the color grouping functionality, the functionality of CFUCounter was compared to that of OpenCFU. However, OpenCFU could not detect white colonies on the plate. The results are displayed in Fig. 6 below.

## Discussion

The enumeration of CFUs on a plate is a crucial yet tedious and error-prone procedure. However, current solutions applied to automate this process are neither convenient nor sufficiently reliable to meet the needs of microbiologists. In this paper, an application is described that easily and conveniently segments and enumerates the cell colonies present on a given plate using only tools commonly found in microbiology laboratories. This application is also capable of grouping colonies by color, opening new avenues for use in various microbiological assays.

As a smartphone-based application, CFUCounter performs adequately in counting colonies and offers high-fidelity segmentation of plate images captured using modern smartphones. Notably, a local minima detector used to generate marker points for watershed segmentation performs reliably well in capturing cell colonies that are affected by the plate edges. This finding represents a progression in the development of CFU counting software because many other solutions opt to reject colonies touching the plate border [18].

CFUCounter also exhibits greater scale invariance than its competitors, as illustrated in Fig. 5a. This is largely attributable to the combination of *a priori* selection and iterative adaptive thresholding, which allow the program to dynamically scale the threshold aperture relative to the selected colony size. In this manner, CFUCounter can consistently produce similar binarized images regardless of the original image size.

Furthermore, CFUCounter demonstrates a high resistance to noise. As presented in Fig. 5b, CFUCounter is still capable of delivering consistent CFU counts despite the colonies being slightly distorted from compression noise. This is attributable to the use of internal standards by CFUCounter rather than hard-coded parameters when conducting parameter tuning. Because the segmentation is affected by every upstream step of the image-processing pipeline, having built-in filters only functions as intended when the conditions match those decided by the programmer. This is not the case for CFUCounter, which can tune its parameters to match the conditions of the image being processed.

CFUCounter also introduces a color grouping functionality, allowing microbiologists to distinguish between the counts of two or more differently colored colonies within a single plate. This is accomplished by integrating multiple color selections from the beginning of the image processing pipeline to the end. Based on the initial number of user selections, CFUCounter generates unique binary masks for each color, which are then used to create a composite mask, allowing for the eventual segmentation and classification of all colonies of interest. The *a priori* selection also informs the number of expected clusters, thereby obviating the necessity for the coarseness slider of OpenCFU.

Prior to this study, local minima detection was used to count colonies; however, it lacked the ability to segment the colonies and distinguish between true and false detections. As such, this approach is extremely noise-sensitive, necessitating the development of a camera and lighting apparatus capable of uniformly capturing the images. Although this simplified approach reduces the time required to analyze a given image, the construction of an imaging device and its inability to reject false detections limit its feasibility and effectiveness.

The adoption of an unsupervised machine learning algorithm to distinguish single-colony detections from multiple colonies and noise also has certain benefits over pure algorithmic and supervised approaches because it should allow for the segmentation of colonies based on the plate-specific colony characteristics. This increases the robustness of the application by allowing the characterization of cells that do not conform to rigid morphological parameters or training data.

Finally, the addition of color grouping functionality allows CFUCounter to be applied to plates containing two or more differently colored colonies. Although this feature is present in OpenCFU, its functionality requires manual parameter tuning and it does not work properly when tested.

However, this approach is limited by its inability to accurately count plates with a high cell density (> 2000 CFU/plate) and by a manual threshold tuning. Although the accurate counting of dense plates is a common challenge for colony counting software, this is also an area where automation is most beneficial. In addition, high-density plates tend to have colonies almost directly on top of one another, making it difficult to detect the local minima for watershed segmentation.

## Conclusions

In conclusion, an efficient and effective CFU counting software with the color differentiation and border colony detection was presented. In its current state, the developed offers a performance on par with, if not better than, leading CFU enumeration programs.

## Data Availability

CFUCounter can be found at https://github.com/louis8765/CFUCounter. Some of the datasets used and/or analyzed during the current study are available from the OpenCFU repository, http://opencfu.sourceforge.net/samples.php.

## List of abbreviations

CFU: Colony forming unit.
C: Constant that is subtracted from the weighted sum of the neighborhood pixels.
s: Size of neighborhood area.
